# A Causal Analysis of Young Adults’ Binge Drinking Reduction and Cessation

**DOI:** 10.3390/ejihpe13050066

**Published:** 2023-05-18

**Authors:** Tyrone C. Cheng, Celia C. Lo

**Affiliations:** 1 School of Social Work, University of Alabama, Little Hall, Tuscaloosa, AL 35401, USA; 2 Peraton, Defense Personnel and Security Research Center, Seaside, CA 93955, USA; celiaclo@yahoo.com

**Keywords:** binge drinking, young adults, social network, racial disparities

## Abstract

Background: This study, using the multiple disadvantage model (MDM), sought to identify factors (disadvantaging social disorganization, social structural, social integration, health/mental health, co-occurring substance use, and substance treatment access factors) in young adults’ binge drinking reduction and cessation in the United States. Methods: We extracted data on 942 young adult binge drinkers (25–34 years, 47.8% female) from the National Longitudinal Study of Adolescent to Adult Health (Add Health), carrying out a temporal-ordered causal analysis, meaning the evaluation of select variables’ impacts on an outcome at a subsequent time. Results: MDM found a relatively high reduction likelihood for non-Hispanic African Americans and respondents with relatively more education. MDM found a relatively low reduction likelihood accompanying an alcohol-related arrest, higher income, and greater number of close friends. Change to nondrinking was found more likely for non-Hispanic African Americans, other non-Hispanic participants having minority ethnicity, older respondents, those with more occupational skills, and healthier respondents. Such change became less likely with an alcohol-related arrest, higher income, relatively more education, greater number of close friends, close friends’ disapproval of drinking, and co-occurring drug use. Conclusions: Interventions incorporating a motivational-interviewing style can effectively promote health awareness, assessment of co-occurring disorders, friendships with nondrinkers, and attainment of occupational skills.

## 1. Introduction

The present study was intended to identify risk and protective factors in the reduction and cessation of binge drinking by young adults in the U.S. In 2019, more than 34% of American adults aged 18–25 and nearly 25% aged 26 or more were binge drinkers. Binge drinking is defined as consuming four or more alcoholic drinks, for females, and five or more alcoholic drinks, for males, on the same occasion, on at least 1 day out of the past 30 days [1]. Binge drinking has been linked to the mental health disorders depression and anxiety [2] and to the physical disorders liver disease, cancer, high blood pressure, and heart disease [3]. It has also been associated with injury-causing vehicular accidents as well as falls and burns. Research also links binge drinking to three forms of violence—homicide, suicide, and intimate partner violence. Furthermore, 30% of annual alcohol poisoning deaths involve binge drinking [4]; indeed, binge drinking is involved in 18,000 alcohol-related deaths per year [2]. Binge drinking is obviously an important public health issue.

### Literature Review

**Figure 1:**
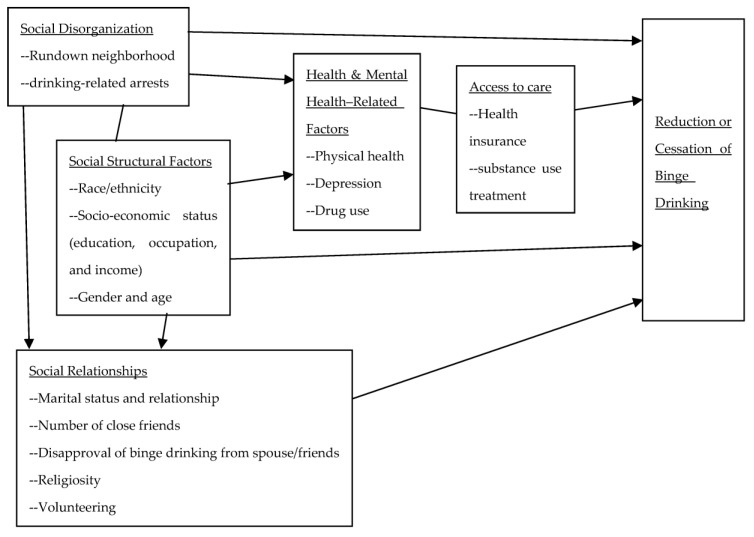
The multiple disadvantage model explaining reduction or cessation of binge drinking.

The multiple disadvantage model has been successfully applied to understand the onset of adult drinking [5], victimization in intimate partner violence [6], and homicide [7,8], as well as access to substance treatment [9]. In the present study, the model tested whether drinking behavior—reduction and cessation, specifically—was linked to six types of disadvantaging factors (see Figure 1). 

First were social-disorganization factors including residence in an unsafe or rundown neighborhood. Next were the three social structural factors: race/ethnicity, income, and education. Next were social integration factors, comprising measures of social relationships and social support. Additionally, we examined health and mental health factors, measures of the respondent’s use of substances beyond alcohol, and factors describing access to substance treatment.

The literature links the social disorganization factor disadvantaged neighborhood, reflecting economic deprivation and multiple social problems, to drinking among adults [5,10,11]. It seems plausible that the challenges of life in disadvantaged neighborhoods could hinder any effort or even desire to give up alcohol. Still, one prior study has reported that living in a disadvantaged neighborhood showed no effect on binge drinking reduction [12]. On the other hand, alcohol-related, possibly binge drinking-related, problems including violence, injury, drunk driving, and property damage clearly contribute to such neighborhoods’ social problems [10]. These risks should logically foster the reduction and cessation of binge drinking [13]. However, the literature notes that binge drinking reduction has been found to diminish where alcohol-related problems are observed [14].

The social structural factors race/ethnicity, income, and education have relationships with binge drinking reduction and/or cessation. A prior study conducted within one state of the United States showed that African Americans and Asian Americans were less likely than White Americans to engage in binge drinking [15]. Compared to White Americans, a different study of binge drinkers found that Americans of a minority ethnicity were less likely to stop binge drinking but more likely to reduce binge drinking [13]. An additional study on pregnant women, showed that respondents of a minority ethnicity were less likely than White respondents to cease or reduce binge drinking [16]. Yet, another study reported no such relationship [17]. Furthermore, one study in the literature reports that binge drinking reduction diminished with higher family income [18], and another that binge drinking reduction and/or cessation increased with additional education [16,17]. We could not locate any prior research about possible relationships between binge drinking reduction and cessation and level of occupational skill.

According to the literature, binge drinking reduction is promoted by social support from family and friends and by the presence of networks of nondrinking relatives and friends [19]. In addition, binge drinking reduction appears to be promoted by being unmarried and also by being a parent [18]. It is reasonable to think, then, that social support, social networks, and marital status might also have impacts on binge drinking cessation. Since participation in religious activities has been observed to cultivate cessation of alcohol use [20,21,22,23], we speculated in the present study that participation in religious or other community-based activities would facilitate cessation and reduction of binge drinking. 

Generally, among adult drinkers, being ill or in poor physical health is linked to cessation of alcohol use [24,25]. Often, binge drinkers with depression cease consuming alcohol only to continue battling depressive symptoms [26]. Some studies in the literature report that depressive clients are relatively less likely to stop drinking [21,27]; other studies found no such relationship [28]. In addition, the literature reports that access to Medicaid facilitates cessation of binge drinking, and yet the lack of such access demonstrates no impact on reduction in binge drinking [16]. Nevertheless, receiving treatment for alcohol use enabled participants in several studies to reduce their binge drinking [29,30,31,32,33,34]. Co-occurring use of other substances, however, hinders the cessation of alcohol use in general [24,27,28].

These many mixed results need to be addressed as research continues. Therefore, we hypothesized, in our study, that among young adults, the reduction and cessation of binge drinking would be associated negatively with social disorganization (i.e., unsafe neighborhood, drinking-related arrest), with physical health, and with drug use. We also hypothesized that these outcomes would be associated positively with social structural factors (i.e., gender, age, race/ethnicity, income, education, occupation) and social integration (i.e., marital status, spousal relationship, close friends, spousal disapproval of binge drinking, close friends’ disapproval of binge drinking, religiosity, volunteering). Finally, we hypothesized that the outcomes would have a positive association with mental health (i.e., depression), access to health insurance (i.e., health insurance coverage), and access to substance treatment (i.e., receipt of counseling).

## 2. Materials and Methods

### 2.1. Sample

The present sample was drawn from the National Longitudinal Study of Adolescent to Adult Health, or Add Health, data set [35]. Between 1994 and 2008, Add Health collected data from over 15,000 nationally representative respondents orchestrated through four waves of interviews. The original core sample’s response rate was 77% at Wave 3 and 78% at Wave 4 [36]. Through in-home interviews in Wave 3 and Wave 4, Add Health researchers collected information on respondents’ substance use, social and economic factors, physical and mental health, family, peers, school, and community [37]. Most respondents were aged 18–26 at Wave 3 (in 2001–2002). Since Add Health collected longitudinal data from a nationally representative sample, such data allows the examination of changes in young adults’ binge drinking behaviors over time. Furthermore, the data set collected information on neighborhoods and on individuals’ alcohol-related arrests. Add Health’s public-use data included only 6504 adolescents in its Wave 1 core sample. To examine possible changes in binge drinking mode over time, we isolated the Wave 3 data of 942 young adult binge drinkers and sought predictors of their drinking reduction or cessation at Wave 4. We defined binge drinker as a respondent who self-reported consuming at least 4 alcoholic drinks, for a female, or at least 5 alcoholic drinks, for a male, on the same occasion, on at least 1 day out of the past 30 days [1]. 

### 2.2. Measures

For our temporal-ordered causal analysis of the extracted data, the outcome variable binge drinking type had 3 categories. Binge drinking maintained indicated that, from Wave 3 to Wave 4, the respondent did not change their binge drinking behavior. Change to non–binge drinking denoted that a respondent had a binge drinker status at Wave 3 but had a non-binge drinker status at Wave 4. Change to nondrinking indicated that a respondent had a binge drinker status at Wave 3 but had a nondrinker status at Wave 4. Binge drinkers, again, were respondents who self-reported consuming at least 4 drinks, if female, or at least 5 drinks, if male, on the same occasion, on at least 1 day out of the past 30 days. Non-binge drinkers were those respondents who reportedly consumed fewer than 4 drinks, if female, or fewer than 5 drinks, if male, in that same time frame. Nondrinkers were those respondents who self-reported no alcohol consumption in the 12 months preceding the interview. 

Temporal-ordered causal analysis is, basically, an examination of the impact of an explanatory variable at one time (here, at Wave 3) upon the outcome variable at a subsequent time (here, at Wave 4). In conducting this temporal-ordered causal analysis, we measured most of our explanatory variables at Wave 3, and measured each variable’s Wave 3-to-Wave 4 difference. A variable’s Wave 3 value constituted its baseline. If an explanatory variable’s wave-to-wave difference had a positive value, that meant its value at Wave 4 had risen above its baseline. If a variable’s wave-to-wave difference had a negative value, that meant its value at Wave 4 had fallen below its baseline. For the Add Health research, there were few explanatory variables that were measured at Wave 3 but not Wave 4. Several explanatory variables, however, were measured solely at Wave 4, because they affected the outcome fairly instantaneously [38]. Our temporal-ordered causal analysis involved measures of ethnicity and gender as time-invariant variables.

Our study involved 4 sets of explanatory variables. The first comprised social disorganization factors. Unsafe neighborhood at Wave 3, a dichotomous (yes/no) variable, indicated whether a Wave 3 Add Health interviewer had perceived a respondent’s neighborhood or surroundings to be unsafe, implying a disadvantaged neighborhood. Unsafe neighborhood difference indicated any Wave 3-to-Wave 4 change in the characterization of respondents’ neighborhoods. Involved in fights at Wave 3 was the number of times in the 12 months preceding the Wave 3 interview that a respondent had engaged in physical fighting because of drinking. Being arrested at Wave 4 was the number of times a respondent had been arrested, per Wave 4 self-report, on a charge of disturbing the peace or driving while impaired or any drinking-related infraction (0 indicating never, 1 indicating once, 2 indicating more than once). 

Our second set of explanatory variables included social structural and demographic factors. A respondent’s socio-economic status was represented by 3 variables: personal income, in USD; education level (1 indicating no high school graduation, 2 indicating high school graduation, 3 indicating some college, 4 indicating undergraduate degree, and 5 indicating graduate school or higher); and occupational skill (0 indicating not in labor force, 1 indicating service worker, 2 indicating operative/farmer, 3 indicating clerical/sales worker, 4 indicating craftsman, 5 indicating professional/manager). Our classification of occupational skill employed a modified version of Rexroat and Shehan’s well-known scale [39]. Personal income difference, education level difference, and occupational skill difference measured Wave 3-to-Wave 4 changes in personal income, education level, and occupational skill, respectively. The demographic variables we considered in our study were male (versus female), age (in years), and ethnicity (non-Hispanic White [the reference group], Hispanic, non-Hispanic African American, other non-Hispanic ethnic minority). 

Our third set of explanatory variables included social integration factors. Being married at Wave 3, a dichotomous (yes/no) measure, denoted if the respondent was married, cohabiting, or romantically/sexually involved with a partner at Wave 3. Being married difference described a change in marriage, cohabitation, or involvement as time elapsed from Wave 3 to Wave 4. The variable satisfactory spousal relationship at Wave 3 rated respondents’ satisfaction with any spouse/partner reported at the Wave 3 interview. Satisfaction was rated on a 5-point Likert scale with responses ranging from 1 (very unsatisfied) to 5 (very satisfied); respondents who were single were assigned 0. Satisfactory spousal relationship at Wave 4 similarly rated respondents’ satisfaction at the Wave 4 interview. It was assessed as a global construct using the sum of seven 5-point Likert scale items (with a Cronbach’s alpha of 0.87). The 7 items were “enjoyed things together”, “handled problems”, “listened to partner”, “expressed affection”, “enjoyed sex life”, “trusted faithful partner”, and “handled finances” [40,41]. Higher scores indicated more satisfactory relationships.

Additionally, the third set of explanatory variables included partner disapproved binge drinking at Wave 3. This variable indicated if, according to the respondent at the Wave 3 interview, the respondent’s spouse or other partner disapproved of binge drinking. The measures were obtained using a 5-point Likert scale ranging from 1 (strongly approved) to 5 (strongly disapproved). Additionally in our third explanatory variables set, number of close friends measured how many persons a respondent reported being at ease with, comfortable talking to about private matters, or confident calling on for help. Add Health researchers specified five responses for the measure: 1 (no close friend), 2 (1–2 close friends), 3 (3–5 close friends), 4 (6–9 close friends), and 5 (10 or more close friends). Number of close friends difference measured any Wave 3-to-Wave 4 change in number of close friends. Close friends disapproved binge drinking at Wave 3 rated close friends’ disapproval of binge drinking, reported at Wave 3. A 5-point Likert scale ranging from 1 (strongly approved) to 5 (strongly disapproved) was used to measure this variable.

Four further explanatory variables made up the third set. Religiosity at Wave 3 represented frequency of engagement in religious activities of any type in the 12 months preceding the Wave 3 interview. Offered responses ranged from 0 (never) to 5 (more than once per week). Religiosity difference denoted Wave 3-to-Wave 4 changes in frequency of religious engagement. The dichotomous (yes/no) variable volunteering noted if a respondent had performed volunteer work or served a community in some way in the 12 months preceding the Wave 3 interview. Volunteering difference tracked any Wave 3-to-Wave 4 change in such work or service of the respondents.

The fourth set of explanatory variables employed in this study presented health, mental health, and healthcare access factors. The respondents self-reported their general physical health at Wave 3 as either 5 (excellent), 4 (very good), 3 (good), 2 (fair), or 1 (poor). General physical health difference measured any change in general health from Wave 3 to Wave 4. Depressive feelings at Wave 3 was measured by the 9-item version of the Center for Epidemiologic Studies Depression Scale (CES-D). The scale yields respondents’ total score on self-reported “feeling depressed”, “losing appetite”, “experiencing hopelessness”, and other symptoms of depression. The same CES-D scale was used to rate each of the 9 symptoms or feelings. The responses for each item ranged from 0 (never/rarely) to 3 (most/all of the time); higher scores indicated more frequent depressive feelings. The scale generated a Cronbach’s alpha of 0.82 at Wave 3 and of 0.79 at Wave 4. Depressive feelings difference denoted any change in the frequency of depressive feelings from Wave 3 to Wave 4. Two dichotomous (yes/no) measures represented healthcare access. Private health insurance at Wave 4 indicated any possession of health coverage through employment, a spouse or parent, union, school, or private purchase. Public health insurance at Wave 4 indicated coverage by Medicaid or the federal Indian Health Service. For this measure, the reference group comprised respondents with no health insurance. Additionally, a dichotomous (yes/no) answer for attended drug treatment at Wave 3 measured respondent self-reports of alcohol or drug treatment received in the 12 months preceding the Wave 3 interview. Attended counseling services at Wave 4 (yes/no) indicated if a respondent had been counseled for a psychological or emotional problem in the 12 months preceding the Wave 4 interview. The final variable in the set, drug use at Wave 4 (yes/no), measured whether a respondent had used marijuana, cocaine, inhalants, or another illicit drug in the 30 days preceding the Wave 4 interview.

### 2.3. Data Analysis

Because our outcome variable had 3 categories and Add Health employed a clustering sample design, our data analysis involved applying Stata software’s version 15.1 survey procedures for multinomial logistic regression (featuring linearized variance estimations of robust standard errors). The analysis used statistically significant relative risk ratios (RRRs) to identify significant predictors of our outcome variable. With the clusters and Wave 4 sampling weights provided by Add Health, we attained a final sample of 129 clusters. Preliminary analysis of tolerance statistics (≥0.49) and correlations (−0.60 ≤ *r* ≤ 0.27) suggested no multicollinearity problems among the explanatory variables.

## 3. Results

Of the 942 binge drinkers in our sample, 44.7% remained binge drinkers at Wave 4, while 46.6% became non-binge drinkers and 8.7% became nondrinkers (see Table 1). 

The average age at Wave 3 was 28.4 years and 52.2% of the sample was male. Non-Hispanic Whites constituted 77.0% of the sample, with non-Hispanic African Americans constituting 9.8%, Hispanics constituting 10.2%, and other non-Hispanic ethnic minorities constituting 3.0%. At Wave 3, 21.2% of the sample were married, 3.0% resided in unsafe neighborhoods, 28.6% volunteered, and 4.4% attended drug treatment. Of the sample at Wave 4, 70.3% had private health insurance, while 5.5% had public health insurance. At Wave 4 as well, 10.3% of the sample attended counseling, and 28.2% self-reported drug use. 

The following are some Wave 3 average measures that we obtained: involved in fights, 0.3 of a possible score of 4.0; personal income, USD 11,125.10 of a possible USD 100,000 income; education, 2.6 (high school graduation); occupational skill, 2.0 (operative/farmer); spousal relationship, 0.4 of a possible 5.0 score; partner disapproved binge drinking, 0.6 of a possible score of 5.0; number of close friends, 2.2 (1–2 close friends); close friends disapproved binge drinking, 3.6 of a possible score of 5.0; religiosity, 1.3 (once to a few times); general physical health, 3.9 of a possible score of 5.0, meaning very good; and depressive feelings, 4.8 of a possible score of 22.0. The Wave 4 average measures were 0.4 (meaning never) for drinking-related arrest in the 12 months preceding the Wave 4 interview and 4.2 of a possible score of 5.0 for satisfaction with spousal relationship at Wave 4.

For seven variables, moving from Wave 3 to Wave 4, we observed increases in average scores. Personal income had an increase of USD 23,344.20. For the variable education difference, the average score rose by 0.6. For the variable occupational skill difference, the average score rose by 1.1. For the variable being married difference, the average score rose by 0.4. In addition, for the variable number of close friends difference, the average score rose by 1.1. Finally, for volunteering difference, the average score rose by 1.1, while for depressive feelings difference, the average score rose by 0.3. In contrast, for two variables, moving from Wave 3 to Wave 4, the average scores showed decreases. The average score for religiosity difference showed a decrease of −0.1, while that for general physical health difference showed a decrease of −0.3. 

### Multivariate Analysis Results 

The results of multinomial logistic regression confirmed that the hypothesized model differed significantly from the null model (*F* = 5.26, *p* < 0.01; see Table 2). 

The likelihood of change to non-binge drinker at Wave 4 was negatively associated with reported arrest at Wave 4 (RRR = 0.62, *p* < 0.01); with personal income difference (RRR = 0.99, *p* < 0.05); with number of close friends at Wave 3 (RRR = 0.18, *p* < 0.01); and with number of close friends difference (RRR = 0.75, *p* < 0.01). Such a likelihood was associated positively with non-Hispanic African American ethnicity (RRR = 2.28, *p* < 0.01); with education level at Wave 3 (RRR = 1.45, *p* < 0.01); and with education level difference (RRR = 1.60, *p* < 0.01). Other variables’ associations with the likelihood of change to non-binge drinker were insignificant.

Eight of the tested factors proved to reduce the likelihood of change to nondrinker at Wave 4. They included self-reported arrest at Wave 4 (RRR = 0.61, *p* < 0.01); relatively high personal income at Wave 3 (RRR = 0.99, *p* < 0.05); relatively more education at Wave 3 (RRR = 0.60 *p* < 0.05); and relatively larger wave-to-wave increase in education (RRR = 0.51, *p* < 0.01). The other four factors were relatively high number of close friends at Wave 3 (RRR = 0.03, *p* < 0.01); relatively large wave-to-wave increase in number of close friends (RRR = 0.56, *p* < 0.01); close friends’ relatively greater disapproval of binge drinking at Wave 3 (RRR = 0.63, *p* < 0.01); and drug use at Wave 4 (RRR = 0.38, *p* < 0.01).

In turn, six of the tested factors increased likelihood of change to nondrinking at Wave 4. They included non-Hispanic African American ethnicity (RRR = 5.16, *p* < 0.01); other non-Hispanic ethnic minority (RRR = 3.22, *p* < 0.05); and relatively older respondents (RRR = 1.45, *p* < 0.01). These six factors also included relatively greater occupational skill (RRR = 1.41, *p* < 0.05); relatively greater wave-to-wave increase in occupational skill (RRR = 1.22, *p* < 0.05); and relatively greater wave-to-wave increase in physical health (RRR = 1.47, *p* < 0.01). The remaining variables showed no significant associations with the likelihood of change to nondrinker at Wave 4.

## 4. Discussion

The 942 binge drinkers constituting our sample made up 24.5% of the 3844 young adults interviewed in Wave 3, which took place during 2001–2002. This percentage of binge drinkers was less than the proportion reported in a prior study [1]. The discrepancy may suggest growth, over the years, in the number of binge drinkers in the young adult population of the U.S. The present study also found a relatively large proportion of binge drinkers, 46.7%, had become non-binge drinkers eventually, although less than 9% were observed to become nondrinkers. In other words, among young adults, becoming a nondrinker is uncommon. Furthermore, almost 45% of binge drinkers in our study maintained their binge drinking behavior. 

The present study’s multivariate results partially supported the hypotheses that (1) binge drinking reduction and cessation is negatively associated with social disorganization (i.e., unsafe neighborhood, drinking-related arrest), with physical health, and with drug use; (2) these outcomes are positively associated with social structural factors (i.e., gender, age, race/ethnicity, income, education, occupation) and social integration (i.e., marital status, spousal relationship, close friends, spousal disapproval of binge drinking, close friends’ disapproval of binge drinking, religiosity, volunteering); and (3) the outcomes have a positive association with mental health (i.e., depression), access to health insurance (i.e., health insurance coverage), and access to substance treatment (i.e., receipt of counseling). In observing no link between binge drinking and unsafe neighborhoods, our study confirms some prior results [12]. Our multivariate results also support other published research that found, as our study did, a negative association between arrest for drinking-related offenses and likelihood of change to non-binge drinker, as well as to nondrinker [14]. Such results imply that, in a vicious circle, binge drinkers may use binge drinking to manage the challenges of residing in disadvantaged neighborhoods whose disadvantages are fueled by the disruptions caused by their binge drinking. It is implied that an effective intervention would be public health education specifically for young adults who live in disadvantaged neighborhoods and have arrest records involving or suggesting typical drinking-related problems. 

The present study showed non-Hispanic African American respondents to be 1.2 times more likely to achieve binge drinking reduction and 4.2 times more likely to achieve drinking cessation than non-Hispanic White respondents; similarly, other non-Hispanic ethnic minority respondents were 2.2 times more likely than non-Hispanic White respondents to achieve drinking cessation. Such results support certain published results [13]. Probable explanations for African Americans’ likelihood of binge drinking cessation included their strong ethnic identity [42] and neighborhood norms of rejection of African American binge drinkers [43]. In contrast, cessation by Asian Americans may be explained by the rejection of binge drinking by some Asian subgroups. It could also be explained by some of these subgroups’ adjustment to their host nation’s cultural discouragement of binge drinking (however, in tandem with acceptance of drinking) [44,45].

Consistent with prior results [16,17], our present findings confirmed that the likelihood of binge drinking reduction was positively associated with education level, as well as with wave-to-wave differences in education level. At the same time, our findings indicated that having more education, as well as increasing attainment of education, were linked to a lower likelihood of drinking cessation. This contradicted the results of a prior study involving solely pregnant women [16]. The present study observed that a relatively high income, as well as an increasing income, diminished the likelihood of drinking cessation and likelihood of binge drinking reduction, respectively. This observation supported results of a prior study [18]. Our study also showed that having more occupational skills as well as improving occupational skills increased the likelihood of drinking cessation. Such results can be plausibly explained. The attainment of relatively more education probably motivates individuals to cut back on binge drinking but not actually cease alcohol consumption. In addition, a growing income increases spending power, including spending on alcoholic beverages. In contrast, growing occupational skill may well be attended by growing responsibility at work, with the employer expectations perhaps facilitating drinking cessation. 

One earlier study with a small sample of Native American women [18] led to results unlike those of our present study, in which marital status, satisfaction with relationship, and partner disapproval of binge drinking showed no links to binge drinking reduction or cessation. In addition, our findings show that religious or volunteer community activities were not associated with binge drinking reduction or cessation. This contradicts prior findings concerning alcohol use in general [20,21,22,23].

In addition, our study found that respondents with more close friends or a growing number of close friends were less likely to reduce binge drinking or experience binge drinking cessation, compared to respondents with few close friends. Moreover, the likelihood of binge drinking cessation was observed to dwindle with relatively strong Wave 3 disapproval of binge drinking by close friends. An implication of such results is that young adults whose binge drinking continues unabated have, and make new, “drinking friends” instead of friends who abstain and also likely disapprove of binge drinking. Our findings in this area underscore the importance of maintaining a network of non-drinking friends [19], regardless of the existence of any spousal partner or religious commitment. 

Our study observed that depressive feelings were not associated with binge drinking reduction or cessation. Furthermore, the absence of any apparent link, in our study, between substance treatment and binge drinking reduction or cessation puts the study at odds with prior work [29,30,31,32,33,34]. We also found that self-reported counseling at Wave 4 was not associated with the outcome, nor was the insurance type.

Unsurprisingly, this study showed that drug use at Wave 4 reduced the likelihood of binge drinking cessation. Additionally, the present study found no association between binge drinkers’ health or improving health and their likelihood of binge drinking reduction; nevertheless, those whose health improved from Wave 3 to Wave 4 were the most likely to give up binge drinking. It seems plausible that many binge drinkers have emotional/behavioral problems that they continue palliating, temporarily, with alcohol or drugs. Only those who come to fully acknowledge alcohol’s role in their health are likely to pursue cessation in order to regain health. These findings imply a need for interventions helping binge drinkers who also use illicit drugs to gain insight on how drug use negatively affects their health and mental health.

Three notable limitations affected our present study. First, the data were based on self-reports and may be susceptible to social desirability bias. Second, the study’s exclusive focus on young adults means its results may not be generalizable to those outside that age group. A third limitation is that Add Health interviewers, not respondents themselves, described the level of safety of the respondents’ neighborhoods. 

## 5. Conclusions

The most important findings of the present study were that alcohol-related arrests, drug use, a relatively higher income, and relatively large network of friends were risk factors that curtailed binge drinking reduction and cessation within this study’s sample of young adults. In contrast, a non-Hispanic minority ethnicity fostered the group’s reduction and cessation of binge drinking. Additionally, the factors education, occupational skill, and friends’ disapproval of drinking affected the reduction and/or cessation of binge drinking.

The present study apparently observed an intertwined group of risk and protective factors characterizing young adults’ binge drinking reduction and drinking cessation that could provide broader implications for interventions and practice. 

To be efficient and successful, our study’s results suggest that any campaign fostering reduction/cessation of binge drinking would need to focus on young adult binge drinkers living in disadvantaged neighborhoods who have been arrested for alcohol-related offenses. The campaign would need to inform the individuals about behaviors that can improve health. It would be essential to reach young adult binge drinkers who use another substance as well as alcohol or who have a dual diagnosis. To curtail binge drinking, interventions should stress expanding clients’ awareness of how one’s alcohol-using friends can influence one’s choices in unhealthy ways. Interventions should foster the development of networks of nondrinking friends. Completing this kind of intervention among college students who are young adults—especially while using a motivational-interviewing style—should prove effective [46]. Interventions provided to young adults of a minority ethnicity should, moreover, promote ethnic identity and explore various cultural beliefs about binge drinking. One further profitable aim of intervention would be to equip young adult binge drinkers with better occupational skills.

Future research similar to ours is needed. The role played by cultural factors like ethnic identity and acculturation in binge drinking reduction/cessation by young non-Hispanic minority adults should be a topic of future studies; so should the role of mental disorders other than depression. Furthermore, research remains to be performed exploring binge-drinking behavior’s relationship to social disorganization factors such as neighborhood norms or racial discrimination. Future research might also ask why education and income demonstrate such opposite effects on the reduction in binge drinking. Future research could, moreover, explore factors in binge drinking reduction and cessation by individuals in later adulthood. 

## Figures and Tables

**Table 1:** Descriptive statistics (n = 942).

Variables		%	Mean	Range	s.d.
Binge drinking type					
Binge drinking maintained		44.7			
Change to non-binge drinking		46.6			
Change to nondrinking		8.7			
Social disorganization factors					
Unsafe neighborhood at Wave 3	(yes)	3.0			
	(no)	97.0			
Unsafe neighborhood difference			0.01	−1–1	0.3
Involved in fights at Wave 3			0.3	0–4	0.7
Being arrested at Wave 4			0.4	0–2	0.5
Social structural and demographic factors					
Personal income at Wave 3			USD 11,125.1	USD 0–1,000,000	USD 10,068.2
Personal income difference			USD 23,344.3	USD −80,000–401,100	USD 31,254.2
Education level at Wave 3			2.6	1–5	0.9
Education level difference			0.6	0–4	0.7
Occupational skill at Wave 3			2.0	0–5	1.2
Occupational skill difference			1.1	−5–5	1.9
Male		52.2			
Female		47.8			
Age (in years)			28.4	25–34	1.6
Non-Hispanic White		77.0			
Hispanic		10.2			
Non-Hispanic African American		9.8			
Other non-Hispanic ethnic minority		3.0			
Social integration factors					
Being married at Wave 3	(yes)	21.2			
	(no)	78.8			
Being married difference			0.4	−1–1	0.6
Satisfactory spousal relationship at Wave 3			0.4	0–5	0.8
Satisfactory spousal relationship at Wave 4			4.2	0–5	0.8
Partner disapproved binge drinking at Wave 3			0.6	0–5	1.5
Number of close friends at Wave 3			2.2	1–3	0.1
Number of close friends difference			1.1	−1–3	1.0
Close friends disapproved binge drinking at Wave 3			3.6	1–5	0.6
Religiosity at Wave 3			1.3	0–5	1.3
Religiosity difference			−0.1	−5–5	1.4
Volunteering at Wave 3	(yes)	28.6			
	(no)	71.4			
Volunteering difference			0.1	−1–1	0.6
Health/mental health/healthcare access factors					
General physical health at Wave 3			3.9	1–5	0.9
General physical health difference			−0.3	−4–4	1.0
Depressive feelings at Wave 3			4.8	0–22	4.2
Depressive feelings difference			0.3	−18–18	4.3
Private insurance at Wave 4	(yes)	70.3			
	(no)	29.7			
Public insurance at Wave 4	(yes)	5.5			
	(no)	94.5			
Attended drug treatment at Wave 3	(yes)	4.4			
	(no)	95.6			
Attended counseling services at Wave 4	(yes)	10.3			
	(no)	89.7			
Drug use at Wave 4	(yes)	28.2			
	(no)	71.8			

Note: s.d. = standard deviation.

**Table 2:** Results of multinomial logistic regression on change to non-binge drinking and change to nondrinking (n = 942).

Variables	Change to Non-Binge Drinking (Binge Drinking Maintained)		Change to Nondrinking (Binge Drinking Maintained)	
	RRR	LSE	RRR	LSE
Social disorganization factors				
Unsafe neighborhood at Wave 3 (no)	1.25	0.72	0.57	0.65
Unsafe neighborhood difference	1.00	0.41	0.55	0.59
Involved in fights at Wave 3	0.83	0.10	0.92	0.21
Being arrested at Wave 4	0.64 **	0.11 **	0.61 **	0.11 **
Social structural and demographic factors				
Personal income at Wave 3	0.99	9.40 × 10^−6^	0.99 *	0.00 *
Personal income difference	0.99 *	2.71 × 10^−6^ *	0.99	7.81 × 10^−6^
Education level at Wave 3	1.45 **	0.17 **	0.60 **	0.11 **
Education level difference	1.60 **	0.20 **	0.51 **	0.14 **
Occupational skill at Wave 3	1.11	0.12	1.41 *	0.25 *
Occupational skill difference	1.05	0.07	1.22 *	0.13 *
Male	0.85	0.17	0.87	0.30
Age (in years)	1.05	0.06	1.45 **	0.13 **
Hispanic (Non-Hispanic White [NHW])	1.06	0.26	1.30	0.63
Non-Hispanic African American (NHW)	2.28 *	0.74 *	5.16 **	3.04 **
Other non-Hispanic ethnic minority (NHW)	1.05	0.64	3.22 *	2.14 *
Social integration factors				
Being married at Wave 3 (no)	0.73	0.19	0.87	0.48
Being married difference	0.91	0.17	1.09	0.41
Satisfactory spousal relationship at Wave 3	1.06	0.11	0.92	0.15
Satisfactory spousal relationship at Wave 4	1.13	0.13	0.89	0.21
Partner disapproved binge drinking at Wave 3	0.95	0.05	1.09	0.11
Number of close friends at Wave 3	0.18 **	0.11 **	0.03 **	0.04 **
Number of close friends difference	0.75 **	0.08 **	0.56 **	0.12 **
Close friends disapproved binge drinking at Wave 3	0.97	0.12	0.63 **	0.12 **
Religiosity at Wave 3	1.03	0.08	1.10	0.17
Religiosity difference	1.06	0.07	1.22	0.15
Volunteering at Wave 3 (no)	1.37	0.31	0.77	0.33
Volunteering difference	1.13	0.19	0.77	0.24
Health/mental health/healthcare access factors				
General physical health at Wave 3	1.03	0.13	0.98	0.22
General physical health difference	1.17	0.13	1.47 **	0.23 **
Depressive feelings at Wave 3	1.02	0.03	1.01	0.05
Depressive feelings difference	0.98	0.02	0.99	0.05
Private insurance at Wave 4 (no)	0.79	0.16	1.47	0.48
Public insurance at Wave 4 (no)	0.56	0.22	1.08	0.61
Attended drug treatment at Wave 3 (no)	1.19	0.53	1.06	0.65
Attended counseling services at Wave 4 (no)	1.02	0.35	1.73	0.75
Drug use at Wave 4 (no)	0.73	0.14	0.38 **	0.12 **
Constant	3.71	8.45	0.49	2.20
*F* =	5.26 **			

Notes: RRR = relative risk ratio; LSE = linearized standard error; * *p* < 0.05; ** *p* < 0.01; reference category or group is indicated in parentheses.

## Data Availability

The Add Health was obtained through Inter-university Consortium for Political and Social Research; www.icpsr.umich.edu (accessed on 22 March 2023).
